# White spot syndrome virus entry is dependent on multiple endocytic routes and strongly facilitated by Cq-GABARAP in a CME-dependent manner

**DOI:** 10.1038/srep28694

**Published:** 2016-07-07

**Authors:** Rong-yuan Chen, Kai-li Shen, Zhen Chen, Wei-wei Fan, Xiao-lu Xie, Chuang Meng, Xue-jiao Chang, Li-bing Zheng, Joseph Jeswin, Cheng-hua Li, Ke-jian Wang, Hai-peng Liu

**Affiliations:** 1 State Key Laboratory of Marine Environmental Science, Xiamen University, Xiamen 361102, Fujian, PR China; 2 Faculty of Life Science and Biotechnology, Ningbo University, 818 Fenghua Road, Ningbo 315211, Zhejiang, PR China; 3 Fujian Collaborative Innovation Center for Exploitation and Utilization of Marine Biological Resources, State-Province Joint Engineering Laboratory of Marine Bioproducts and Technology, Xiamen 361102, Fujian, PR China

## Abstract

White spot syndrome virus (WSSV) is a lethal pathogen of shrimp and many other crustaceans, including crayfish. However, the molecular mechanism underlying its cellular entry remains elusive due to the lack of shrimp cell lines for viral propagation. Crayfish hematopoietic tissue (Hpt) cell culture was recently established as a good model for WSSV infection study. Here, we showed that multiple endocytic routes, including clathrin-mediated endocytosis (CME), macropinocytosis and caveolae-mediated endocytosis, were indispensably employed for the viral entry into Hpt cell of the crayfish *Cherax quadricarinatus*. Intriguingly, cellular autophagic activity was positively correlated with efficient viral entry, in which a key autophagy-related protein, γ-aminobutyric acid receptor-associated protein (Cq-GABARAP), that not only localized but also co-localized with WSSV on the Hpt cell membrane, strongly facilitated WSSV entry by binding to the viral envelope VP28 in a CME-dependent manner that was negatively regulated by Cq-Rac1. Furthermore, cytoskeletal components, including Cq-β-tubulin and Cq-β-actin, bound to both recombinant rCq-GABARAP and WSSV envelope proteins, which likely led to viral entry promotion via cooperation with rCq-GABARAP. Even under conditions that promoted viral entry, rCq-GABARAP significantly reduced viral replication at an early stage of infection, which was probably caused by the formation of WSSV aggregates in the cytoplasm.

White spot syndrome virus (WSSV), the only member of the genus *Whispovirus* in the novel *Nimaviridae* family, is a large enveloped virus (~70–167 nm × 210–380 nm) with double-stranded DNA (~300 kb). WSSV is a lethal pathogen that infects shrimp and many other crustaceans, such as crayfish, crab and lobster. Over the past three decades, extensive studies have been performed to expand our knowledge of WSSV genomics, proteomics and morphogenesis^1^. Increasing numbers of virus-binding proteins involved in viral infection have been described^2^. For example, some cellular surface proteins, such as glucose transporter 1^3^,^4^, calreticulin and C-type lectins^5^, have been proposed to be necessary for WSSV entry. With the use of chemical inhibitors, two recent studies discovered that an endocytic pathway, probably caveolae-mediated endocytosis, is exploited for WSSV entry into hemocytes in both shrimp and crayfish^6^,^7^. Based on transmission electron microscopy (TEM) and endocytosis inhibition assays, Yang and his colleagues recently reported that WSSV enters hematopoietic tissue (Hpt) cells via clathrin-mediated endocytosis in the red claw crayfish *Cherax quadricarinatus,* and the progeny viruses are produced only in Hpt cell but not hemocyte^8^,^9^. It is known that the hemocytes are originally developed in the Hpt and are finally released from this tissue into the blood circulation in crayfish^10^,^11^. Furthermore, a later study proposed a model of WSSV replication in primary cell cultures from the lymphoid organ of *Litopenaeus vannamei*, in which the viral replication cycle is approximately 12 h, and progeny virions are produced and released at the final stage of infection^12^. Together, these findings have advanced our understanding of WSSV infection. However, more details concerning the molecular mechanism underlying WSSV entry into host cells, particularly the molecular events that occur during the early stage of infection, remain largely elusive.

It is well known that viral entry into the host cell is the first step towards a successful infection. Notably, numerous viruses favor endocytosis, such as clathrin-mediated endocytosis (CME), macropinocytosis, caveolae-mediated endocytosis and several clathrin- and caveolin/raft-independent mechanisms^13^, for successful entry. As one of the most well-documented endocytic pathways, CME is characterized by the uptake of cargo, such as the transferrin-iron complex, epidermal growth factor, toxins and viruses, by invaginations or clathrin-coated vesicles^14^. The invaginations are triggered by cargo-receptor binding and the subsequent recruitment of clathrin-associated adaptor protein complexes (AP2), which attach to the membrane via interactions with the endocytic motifs present in the cytoplasmic tails of the receptors^15^ and with phosphatidylinositol-4, 5-bisphosphate (PI(4,5)P2)^16^. Synaptojanin 2, a member of the 5-phosphatase family, regulates CME by modulating the level of PI(4,5)P2 on the cell membrane^17^ to alter the initiation and localization of endocytic membrane trafficking. Rho family proteins, including Rac1, Cdc42 and RhoA, have also been found to regulate this process^18^. For example, activated Rac1 inhibits receptor internalization^19^ through CME by targeting synaptojanin 2^20^. As a key member of the Atg8 family, γ-aminobutyric acid receptor-associated protein (GABARAP) is a well-known ubiquitin-like protein involved in autophagosome biogenesis and intracellular trafficking^21^. The binding of GABARAP to the transferrin receptor^22^ or clathrin heavy chain (CHC)^23^ strongly suggests that GABARAP functions in regulating the CME pathway, of which the mechanism may include that GABARAP is implicated in Rac1 signaling by interacting with or modulating the upstream elements: guanine exchange factors^20^,^24^. To our knowledge, there is no literature concerning virus internalization regulated by GABARAP via modulating CME pathway at present.

Due to the lack of shrimp cell lines for the viral propagation and genetic manipulation of WSSV, current understanding of the pathogenesis of WSSV remains limited. For example, the viral entry mechanism has not been fully elucidated. Crayfish Hpt cell cultures were recently demonstrated to be a good model for investigating the mechanism of WSSV infection^25^, particularly for the successful generation of the progeny WSSV^10^. Therefore, research on WSSV infection in Hpt cell will likely shed new light on the pathogenesis of this lethal virus in aquaculture. In our previous differential screening study, some genes related to endocytosis and autophagy, such as the clathrin light chain (Cq-CLC, GenBank accession no. JF284465.2) and Cq-GABARAP (GenBank accession no. JF284569.2), were isolated from Hpt cell of the red claw crayfish, which further benefits the study of the molecular mechanism underlying WSSV entry into host cells. In this study, we focused on investigating the mechanism through which WSSV entered Hpt cell based on TEM combined with different pharmacological inhibitor assays for blocking different endocytic pathways, including CME, macropinocytosis and caveolae-mediated endocytosis, and autophagy. The role of CME and autophagy in WSSV entry was further validated by gene silencing of the key components of CME, such as Cq-CLC, Cq-AP50 (medium subunit of clathrin-associated adaptor protein complexes) (GenBank accession no. KP698211) and Cq-dynamin (GenBank accession no. KP782023), and the key autophagy factor Cq-GABARAP. Furthermore, the impact of Cq-GABARAP on the regulation of WSSV entry into Hpt cell was also investigated with regard to its possible roles in autophagy, regulation of CME activity, interactions with both virion and Hpt cell, and subsequent effects on viral replication of the internalized viruses.

## Results

### WSSV entered crayfish Hpt cell via multiple endocytic routes

**Figure 1:**
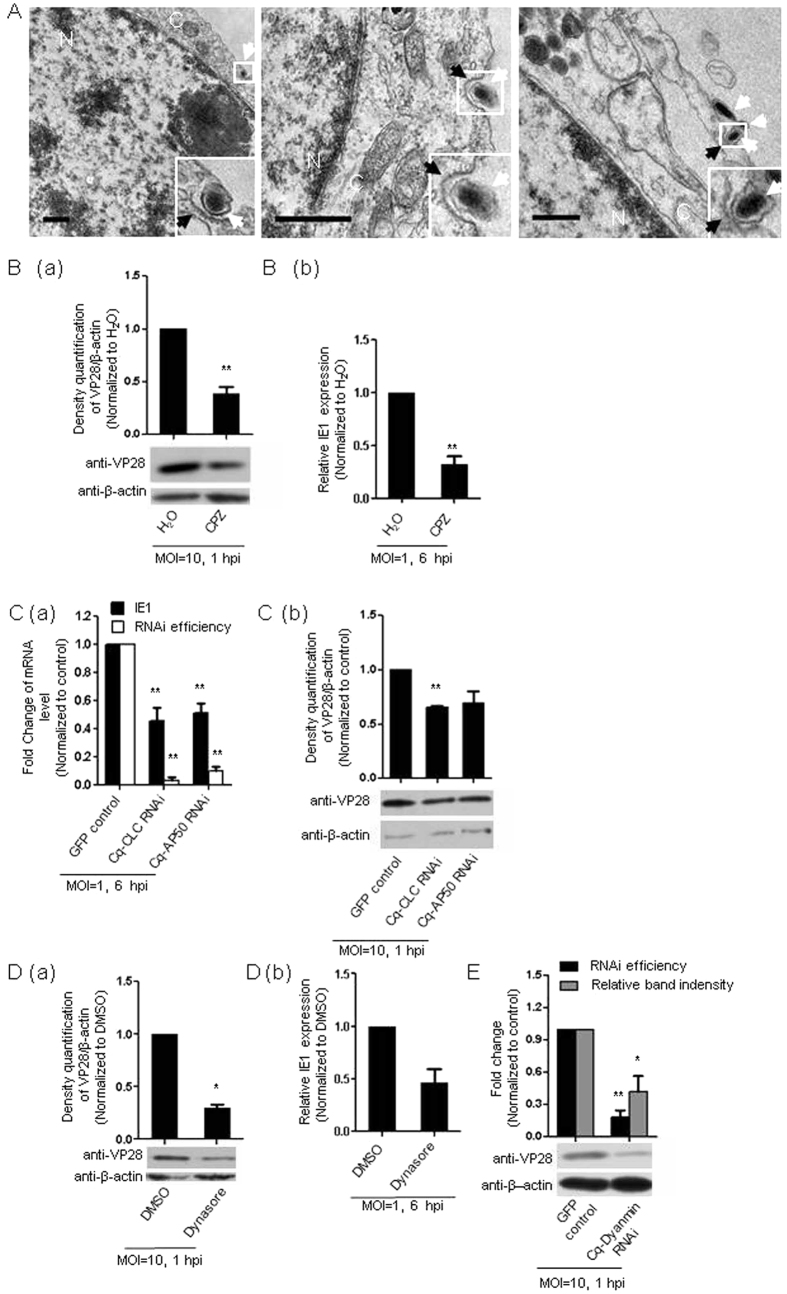
WSSV entry into Hpt cell via CME. (**A**) WSSV virions captured in cell invaginations resembling clathrin-coated pits at 0.5 hpi by TEM. Black arrows indicate clathrin-coated pits, and white arrows indicate WSSV virions. The bars indicate 0.5 μm. C: cytoplasm; N: nucleus. (**B**) Inhibition of WSSV entry by pretreatment of the cells with CPZ. (a) The major viral envelope protein VP28 was immunoblotted with a monoclonal antibody against VP28 at 1 hpi (lower panel). (b) Suppression of WSSV replication by pretreating the cells with CPZ. Suppression was evaluated by assessing relative IE1 gene expression at 6 hpi using qRT-PCR. (**C**) (a) Inhibition of WSSV replication via knockdown of Cq-CLC or Cq-AP50 as assessed by qRT-PCR at 6 hpi to quantify the relative IE1 gene expression. The RNAi efficiency of Cq-CLC and Cq-AP50 was also examined by qRT-PCR. (b) Inhibition of WSSV entry via knockdown of Cq-CLC or Cq-AP50 as determined by immunoblotting against the viral envelope protein VP28 at 1 hpi (lower panel). (**D**) (a) Suppression of WSSV entry by pretreating the cells with dynasore determined by immunoblotting against the viral envelope protein VP28 at 1 hpi (lower panel). (b) Inhibition of WSSV replication by pretreating the cells with dynasore to assess the relative IE1 gene expression by qRT-PCR at 6 hpi. E: RNAi efficiency of Cq-dynamin examined by qRT-PCR (dark bars in upper panel). The suppression of WSSV entry via gene knockdown of Cq-dynamin was determined from the relative quantification of the viral envelope protein VP28 at 1 hpi by immunoblotting (lower panel). The band intensities of three independent experiments were calculated using the Quantity One program (upper panel in **B**(a), **C**(b), **D**(a) and gray bars in upper panel in E). All the results were observed in at least three independent experiments, and one of the representative results is shown. The data are presented as the mean ± SEM from at least three independent experiments and were analyzed by Student’s *t* test (*P < 0.05, **P < 0.01).

TEM was employed to visualize the mechanism by which WSSV entered crayfish Hpt cell. As shown in Fig. 1A, the WSSV particle was captured in a typical clathrin-coated structure with an electron-dense coat along the cytosolic side of the cell membrane^26^, indicating that clathrin-coated pits were probably an important portal for WSSV entry into crayfish Hpt cell. To assess this hypothesis, we employed the pharmacologic inhibitor chlorpromazine (CPZ), a specific inhibitor commonly used for blocking clathrin-mediated endocytosis via interruption of the assembly of clathrin-coated pits on the cell membrane^27^, to suppress the CME pathway. As indicated in Fig. 1B(a), CPZ treatment resulted in a significant decrease in the internalized WSSV as determined by immunoblotting for the WSSV envelope protein VP28, which can not be regarded as the newly synthesized protein until 3 hpi (Supplementary Fig. 1). Therefore, this readout, when detected at 1 hpi, could be used as a good index for the amount of internalized virus. Furthermore, the Hpt cells pretreated with CPZ exhibited a 70% reduction in the expression of the IE1 gene (Fig. 1B(b)), an immediate early gene that was indispensable for initiating WSSV replication^28^. This finding implied that CPZ pretreatment significantly suppressed viral replication. The well-known key components of the clathrin-coated pits, i.e., CLC and AP50, played a vital role during the formation of these pits^14^. To further confirm that CME was involved in WSSV invasion, we examined the amount of entered WSSV virions after gene silencing of the Cq-CLC or Cq-AP50 gene by RNAi. The gene-silencing efficiency was determined by qRT-PCR, which showed the efficient knockdown of these two genes (Fig. 1C(a)). As shown in Fig. 1C(b), the amount of the intracellular VP28 protein was reduced by approximately 50% after silencing of the Cq-CLC or Cq-AP50 gene, indicating that the knockdown of Cq-CLC or Cq-AP50 could inhibit WSSV invasion into Hpt cell. In addition, the viral gene transcription was also determined, and silencing of either Cq-CLC or Cq-AP50 resulted in an approximately 55% reduction of IE1 transcript expression (Fig. 1C(a)). These results demonstrated that Cq-CLC and Cq-AP50, the key components of the CME pathway, were essential for WSSV entry in crayfish Hpt cell, implying that CME played an important role in WSSV entry into host cells. Because dynamin, a well-known small GTPase, plays a key role in endocytic vesicle scission^14^,^29^, we also suppressed dynamin activity using an inhibitor, dynasore, and found a clear decrease in WSSV infection as shown both through the levels of viral internalization and transcription (Fig. 1D(a,b)). Moreover, gene silencing of Cq-dynamin by RNAi also resulted in reduced levels of internalized WSSV in Hpt cell (Fig. 1E) as determined by the above-mentioned analysis of the viral envelope protein VP28, suggesting that dynamin was also involved in WSSV entry into Hpt cell. Taken together, our data provided the first strong evidences that CME played an important function in WSSV entry into host cell at the molecular level.

**Figure 2:**
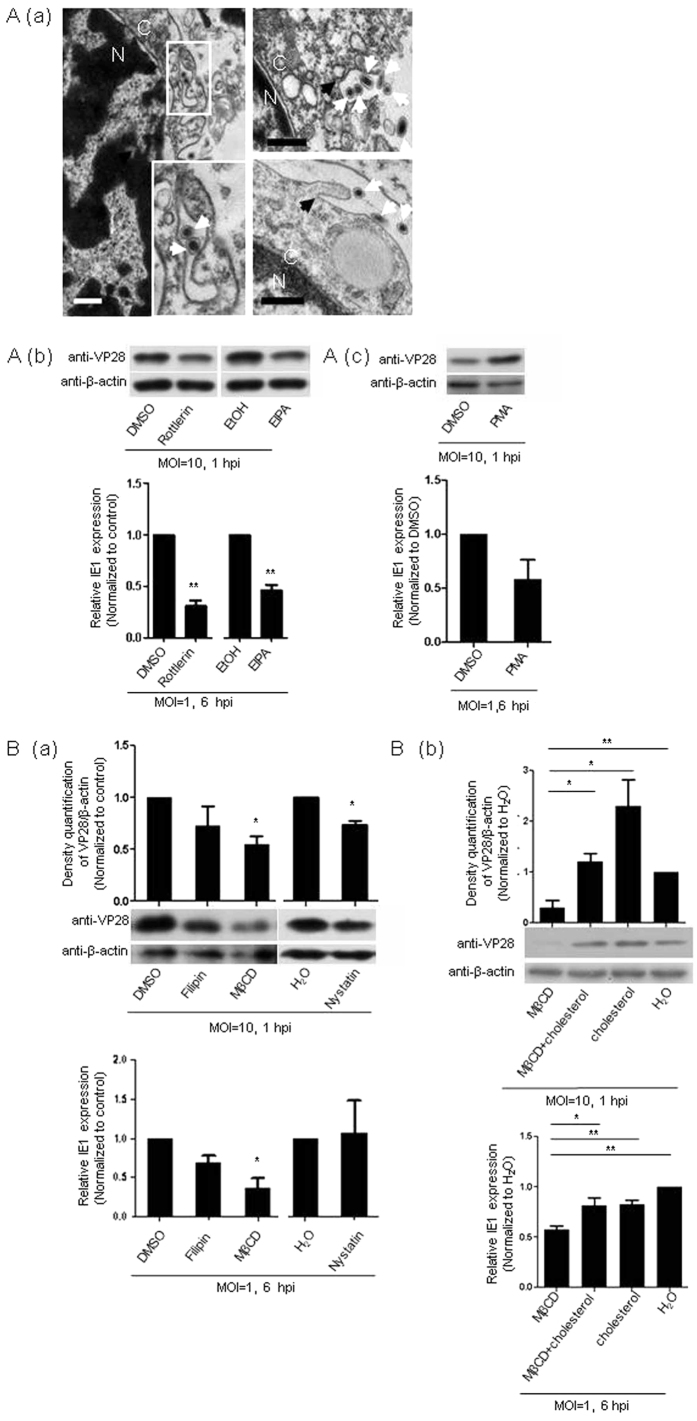
WSSV entry into Hpt cell via alternative endocytic routes. (**A**) WSSV entry into Hpt cell via macropinocytosis. (a) WSSV virions captured by cell protrusions visualized by TEM. Black arrows indicate plasma membrane protrusions or macropinocytic cups, and white arrows indicate WSSV virions. The bars indicate 0.5 μm; C: cytoplasm; N: nucleus. (b) Inhibition of WSSV entry by pretreating the cells with the macropinocytosis inhibitors rottlerin and EIPA assessed by the relative quantification of the viral envelope protein VP28 at 1 hpi (upper panel). WSSV replication was determined by the relative quantification of IE1 gene expression using qRT-PCR at 6 hpi (lower panel). (c) Promotion of WSSV entry by pretreating the Hpt cell with the macropinocytosis inducer PMA determined by immunoblotting to quantify VP28 at 1 hpi (upper panel). WSSV replication was determined by the relative quantification of IE1 gene expression using qRT-PCR at 6 hpi (lower panel). (**B**) WSSV entry into Hpt cell via caveolae-mediated endocytosis. (a) Effect on WSSV entry induced by pretreating the cells with the caveolae-mediated endocytosis inhibitors filipin, MβCD or nystatin as determined by the relative quantification of the viral envelope protein VP28 via immunoblotting at 1 hpi. WSSV replication was determined by the relative quantification of IE1 gene expression using qRT-PCR at 6 hpi (lower panel) after pretreatment with filipin, MβCD or nystatin. (b) Rescue of WSSV entry achieved by cholesterol replenishment following inhibition by MβCD as determined by relative quantification of the viral envelope protein VP28 via immunoblotting at 1 hpi. Rescue of WSSV entry was achieved by cholesterol replenishment following inhibition by MβCD as determined by the relative quantification of IE1 gene expression using qRT-PCR at 6 hpi (lower panel). The band intensities of VP28 and β-actin from three independent experiments were analyzed using the Quantity One program (upper panel in **B**(a,b). All of the results were observed in at least three independent experiments, and one of the representative results is shown. The data are presented as the mean ± SEM from at least three independent experiments and were analyzed using Student’s *t* test (*P < 0.05, **P < 0.01).

Besides, our TEM analysis clearly showed that the WSSV virions were surrounded by cell protrusions or macropinocytic cups (Fig. 2A(a)), typical features of macropinocytosis^30^, suggesting that macropinocytosis was likely involved in WSSV entry into Hpt cell. This speculation was then supported by the finding that both viral entry and replication were suppressed in Hpt cell after the cells were pretreated with the specific macropinocytosis inhibitors 5-(N-ethyl-N-isopropyl)-amiloride (EIPA) or rottlerin (Fig. 2A(b)). In contrast, viral entry was clearly increased if the cells were pretreated with the macropinocytosis inducer phorbol myristate acetate (PMA)^30^, even though no obvious increase in viral gene replication was detected in the presence of PMA (Fig. 2A(c)). Thus, these results provided the first indication that macropinocytosis might serve as an efficient alternate route that was employed for the entry of WSSV into the host cell.

As a special class of cholesterol-containing lipid rafts, caveolae are essential for the structure and function of caveolae-mediated endocytosis^31^. methyl-β-cyclodextrin (MβCD), filipin and nystatin are specific pharmacological inhibitors that are commonly used to interfere with cholesterol and thus caveolae-mediated endocytosis. We found that pretreatment of Hpt cell with MβCD before WSSV inoculation clearly suppressed both WSSV entry and viral replication, while pretreatment with filipin had a slight suppressive effect on viral entry and replication (Fig. 2B(a)). In contrast, nystatin significantly inhibited WSSV entry, even though no obvious effect on WSSV replication was observed at the transcript level (Fig. 2B(a)). To further examine whether these suppressive effects were caused by disruption of the cholesterol in the cellular lipid rafts, the Hpt cells were incubated with additional cholesterol to restore the membrane cholesterol after its reduction by pretreating the cells with MβCD. The results clearly showed that cholesterol replenishment successfully rescued the MβCD-induced inhibition of WSSV entry as well as viral replication (Fig. 2B(b)). In regarding to the crucial component of caveolae in facilitating WSSV invasion, these data revealed an essential role of cholesterol, and an optional route via caveolae-mediated endocytosis was necessary for WSSV entry into crayfish Hpt cell.

### Cellular autophagic activity was positively correlated with WSSV entry

**Figure 3:**
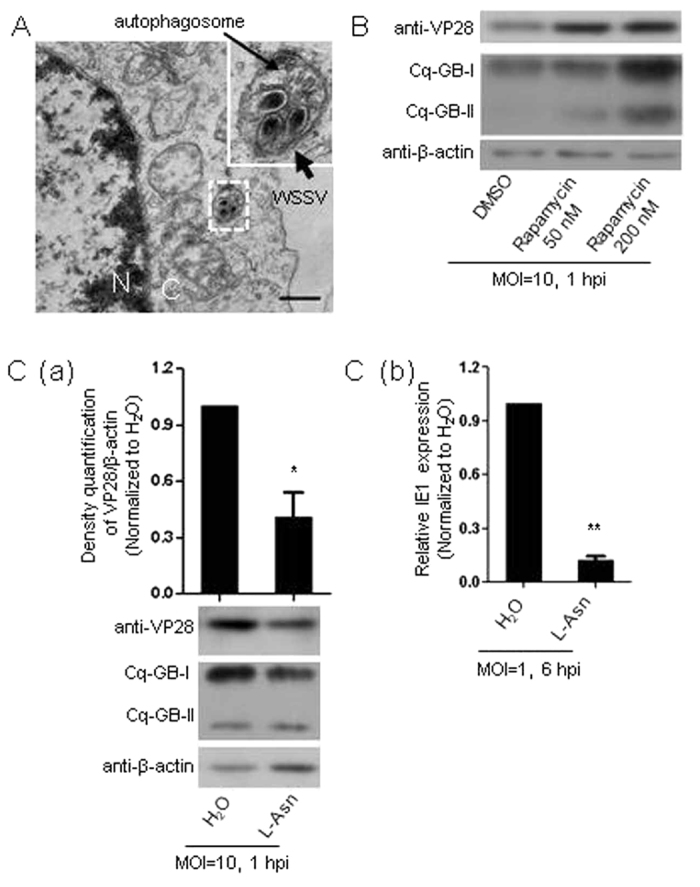
WSSV entry into Hpt cell was positively correlated with cellular autophagic activity. (**A**) WSSV virions localized to autophagosomes visualized by TEM at the early stage of infection (0.5 hpi). The bar indicates 0.5 μm; C: cytoplasm; N: nucleus. (**B**) Increased WSSV entry with induction of autophagic activity by rapamycin. WSSV entry was determined by immunoblotting to quantify the viral envelope protein VP28 at 1 hpi. The induction of autophagy was monitored by detecting the conversion of Cq-GABARAP-I/Cq-GABARAP-II (i.e., Cq-GB-I/Cq-GB-II) with immunoblotting against Cq-GABARAP. (**C**) (a) Reduced WSSV entry and conversion of Cq-GB-I/Cq-GB-II by the autophagy inhibitor L-Asn determined by immunoblotting (lower panel). The band intensities of VP28 and β-actin from three independent experiments were analyzed using the Quantity One program (upper panel). (b) Reduced WSSV entry by L-Asn treatment determined by the relative quantification of IE1 gene expression using qRT-PCR at 6 hpi. All of the results were observed in at least three independent experiments, and one of the representative results is shown. The data are presented as the mean ± SEM from at least three independent experiments and were analyzed by Student’s *t* test (*P < 0.05, **P < 0.01).

Our TEM analysis of viral entry clearly showed that the WSSV virions were enclosed by double-membrane vesicles resembling typical autophagosomes in the cytoplasm (Fig. 3A). We then assessed the effect of autophagy on WSSV entry by changing the autophagic activity of Hpt cell through induction or inhibition with pharmacological chemicals before viral challenge. Interestingly, pretreating the Hpt cell with the autophagy inducer rapamycin obviously increased virion entry (Fig. 3B). In contrast, pretreatment of the Hpt cell with the autophagy antagonist L-asparagine (L-Asn) reduced both WSSV entry and viral replication (Fig. 3C(a,b)). Together, these results provided the first strong indication that cellular autophagic activity was positively correlated with the efficiency of WSSV entry into the host cells at an early stage of infection.

**Figure 4:**
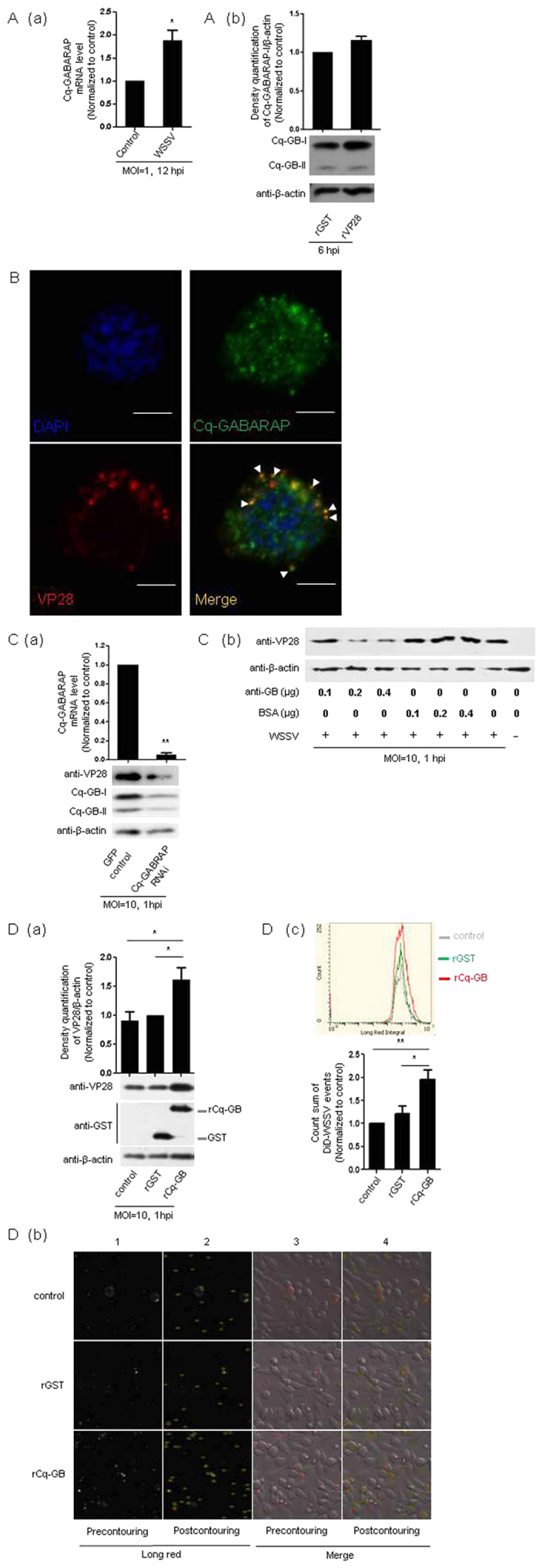
WSSV entry into Hpt cell promoted by rCq-GABARAP. (**A**) Expression of the Cq-GABARAP gene (a) and protein (b) in response to WSSV infection or mock infection with recombinant viral VP28 (rVP28 with a GST tag) as examined by qRT-PCR and immunoblotting, respectively. (**B**) Co-localization of Cq-GABARAP with VP28 in Hpt cell at 3 hpi determined by dual-IFA with anti-GABARAP and anti-VP28 antibody. The nucleus was stained with DAPI. White arrowheads indicate the co-localizations of Cq-GABARAP with VP28. Bars, 5 μm. (**C**) Suppression of WSSV entry via loss-function-of Cq-GABARAP determined by immunoblotting. (a) The RNAi efficiency of Cq-GABARAP was examined by qRT-PCR (upper panel) (b) Hpt cells were transfected with an anti-GABARAP antibody using PULSin before viral infection. The same concentrations of BSA were used as control treatments accordingly. (**D**) WSSV entry promoted by rCq-GABARAP. (a) Enhanced WSSV entry was achieved after pre-incubation of the virus with 2 μg of rCq-GABARAP (rCq-GB) for 30 min determined by immunoblotting for VP28 at 1 hpi (lower panel). (b) Enhanced WSSV entry by rCq-GABARAP examined with a laser-scanning cytometer. DiD-labeled WSSV was incubated with 2 μg of rCq-GABARAP for 30 min and then inoculated into the Hpt cells. Images of the DiD-WSSV-infected cells were obtained using an iCys laser-scanning cytometer at 3 hpi. DiD-WSSV was automatically identified by the iCys software through proper contouring defined by the edge of the virion (green cycle) and pseudocolored with red in panel 3 and 4. (c) DiD-WSSV was identified by contouring (as indicated in panel 2 and 4 in (b)) with an iCys software and was then quantified (upper panel). The sum of DiD-WSSV counts shown in (b) was calculated (lower panel). The band intensities of three independent experiments were calculated using the Quantity One program (upper panel in **A**(b), **D**(a)). All of the results were observed in at least three independent experiments, and one of the representative results is shown. The data are presented as the mean ± SEM from at least three independent experiments and were analyzed by Student’s *t* test (*P < 0.05, **P < 0.01).

The well-known ubiquitin-like protein Atg8 and its homologs, such as GABARAP (designed as Cq-GABARAP in the present study (Supplementary Fig. 2A,B)), localize to autophagosomes, where they are necessary for membrane expansion and completion of the autophagosome^21^. During autophagy, GABARAP is explicitly recruited via increased conversion of GABARAP-I to GABARAP-II, which is a well-known indicator of increased autophagosome formation and thus enhanced autophagic activity^32^. Meanwhile, our previous study has shown that the Cq-GABARAP transcript was up-regulated in Hpt cell after WSSV challenge^33^. In the present study, infection with WSSV or mock infection with the recombinant viral envelope protein rVP28 was further found to increase the expression of the Cq-GABARAP transcript (~2-fold) or protein (~1.2-fold), respectively (Fig. 4A(a,b)). It has been found that LC3, another Atg8 homolog, could additionally localize to the cell membrane and be used by the influenza A virus to maintain virion stability^34^. This led us to determine the distribution of the Cq-GABARAP protein showing that it was widely present in different cellular compartments, including cell membrane, cytoplasm and nucleus in Hpt cell (Supplementary Fig. 2C). Importantly, Cq-GABARAP protein not only localized on the cell membranes (Supplementary Fig. 2D) but also co-localized with the viral envelope protein VP28 in both cell membranes and cytoplasm as determined by confocal microscopy analysis (Fig. 4B), suggesting that Cq-GABARAP itself might function in viral entry since it interacted with WSSV virions on the cell membrane at an early stage of infection. Naturally, we wondered whether Cq-GABARAP, due to its key role in autophagy, directly affected WSSV entry. To address this question, we performed RNAi assays against the Cq-GABARAP gene followed by WSSV challenge and found that knockdown of the Cq-GABARAP gene clearly suppressed the viral entry (Fig. 4C(a)). At the same time, WSSV entry was also decreased if intracellular Cq-GABARAP protein activity was blocked via antibody transfection against endogenous Cq-GABARAP (Fig. 4C(b)), suggesting a vital role for Cq-GABARAP in WSSV entry. To further explore the correlation between Cq-GABARAP and WSSV entry, we then analyzed the efficiency of viral entry by pre-incubating the virions with extra rCq-GABARAP followed by inoculation of the mixture into Hpt cell cultures. Intriguingly, pre-incubation of the rCq-GABARAP protein with WSSV markedly promoted viral entry compared with the control treatment using a GST recombinant protein (Fig. 4D(a)). This observation was also confirmed by a cell imaging tracking assay using an iCys laser-scanning cytometer, which provided images of Hpt cell infected with 1,1′-dioctadecyl-3,3,3′,3′-tetramethylindodicarbocyanine (DiD) labeled DiD-WSSV (panel 1 & 3 in Fig. 4D(b)) and calculated the viral entry events by identifying the DiD fluorescent signal (long red) through program contouring (panel 2 & 4 in Fig. 4D(b,c)) with the iCys software. These data strongly demonstrated that Cq-GABARAP necessarily participated in the WSSV entry into the crayfish Hpt cell.

### rCq-GABARAP-mediated WSSV entry was dependent on CME, negatively regulated by Cq-Rac1, and positively correlated with cellular autophagic activity

**Figure 5:**
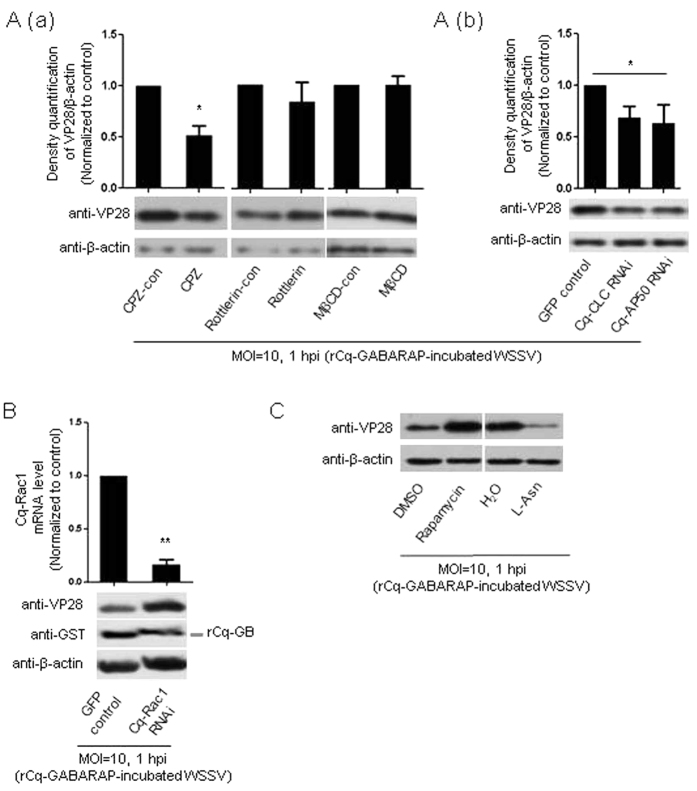
Enhanced WSSV entry mediated by rCq-GABARAP was dependent on CME and positively correlated with autophagy. (**A**) WSSV entry promoted by rCq-GABARAP in a CME-dependent manner. (a) Hpt cells were pretreated with CPZ (2.5 μM), MβCD (500 μM), rottlerin (1 μM) or solvent controls for 30 min followed by infection accordingly with WSSV pre-incubated with rCq-GABARAP. The cells were then harvested for immunoblotting against the viral envelope protein VP28 at 1 hpi (lower panel). The band intensities of three independent experiments were calculated using the Quantity One program (upper panel). (b) Hpt cells were pretreated with dsRNA of Cq-CLC, Cq-AP50 or GFP followed by infection with WSSV pre-incubated with rCq-GABARAP accordingly and then harvested for immunoblotting against VP28 at 1 hpi (lower panel). The band intensities of three independent experiments were calculated using the Quantity One program (upper panel). (**B**) Promotion of rCq-GABARAP-mediated WSSV entry by gene knockdown of Cq-Rac1. The RNAi efficiency of Cq-Rac1 was examined by qRT-PCR (upper panel). WSSV entry was examined by immunoblotting against the viral envelope protein VP28 (lower panel). (**C**) rCq-GABARAP-mediated WSSV entry positively correlated with cellular autophagy activity. The cellular autophagy activity was changed by pretreating Hpt cell with an autophagy inducer (rapamycin, 50 nM) or inhibitor (L-Asn, 30 mM). WSSV entry was assessed by immunoblotting against the viral envelope protein VP28. The data are presented as the mean ± SEM from at least three independent experiments and were analyzed using Student’s *t* test (*P < 0.05).

We previously determined that WSSV entry into Hpt cell depended on at least three endocytic routes: CME, macropinocytosis and caveolae-mediated endocytosis. We then examined the relationship between Cq-GABARAP and these three different endocytic routes with regard to the efficiency of WSSV entry. By combining the treatments with pharmacological inhibitors and rCq-GABARAP, the rCq-GABARAP-mediated enhancement of WSSV entry was clearly suppressed when Hpt cells were pretreated with CPZ (specific inhibitor of the CME pathway), but was apparently not affected by the other tested inhibitors, such as rottlerin (specific inhibitor of macropinocytosis) and MβCD (inhibitor of caveolae-mediated endocytosis) (Fig. 5A(a)). Similarly, the rCq-GABARAP-mediated enhancement of WSSV entry was also suppressed by the gene silencing of Cq-AP50 or Cq-CLC by RNAi (Fig. 5A(b)). Together, these results strongly implied that rCq-GABARAP-promoted WSSV entry into Hpt cell was dependent on CME.

GABARAP is able to promote internalization of the transferrin receptor, the well-known indicator of classic endocytosis, through the CME pathway by abolishing negative regulation via the Rac1/Ost-III signaling pathway in HeLa cells^20^. To determine whether the Cq-GABARAP-mediated enhancement of WSSV entry was related to the Rac1 pathway, we cloned the Cq-Rac1 gene (GenBank accession no. KP765446) from Hpt cell and performed gene silencing of Cq-Rac1 before determination of viral entry. Interestingly, knockdown of the Cq-Rac1 gene enhanced the promoted WSSV entry by rCq-GABARAP (Fig. 5B), suggesting that the Rac1 signaling pathway negatively regulated rCq-GABARAP-promoted WSSV entry in a CME-dependent manner.

Because GABARAP is an important autophagy-related factor^32^ and cellular autophagic activity was positively correlated with WSSV entry into Hpt cell as described above (Fig. 3), we then examined whether autophagy modulators also affect rCq-GABARAP-promoted WSSV entry. As shown in Fig. 5C, significantly less rCq-GABARAP-mediated WSSV entry was observed in the Hpt cell pretreated with the autophagy inhibitor L-Asn. In contrast, pretreatment of the Hpt cell with the autophagy inducer rapamycin increased rCq-GABARAP-mediated WSSV entry (Fig. 5C). Hence, these data indicated that rCq-GABARAP-promoted WSSV entry was also positively correlated with cellular autophagic activity.

### rCq-GABARAP enhanced WSSV entry via binding to the viral envelope protein VP28 and cytoskeleton of Hpt cell

**Figure 6:**
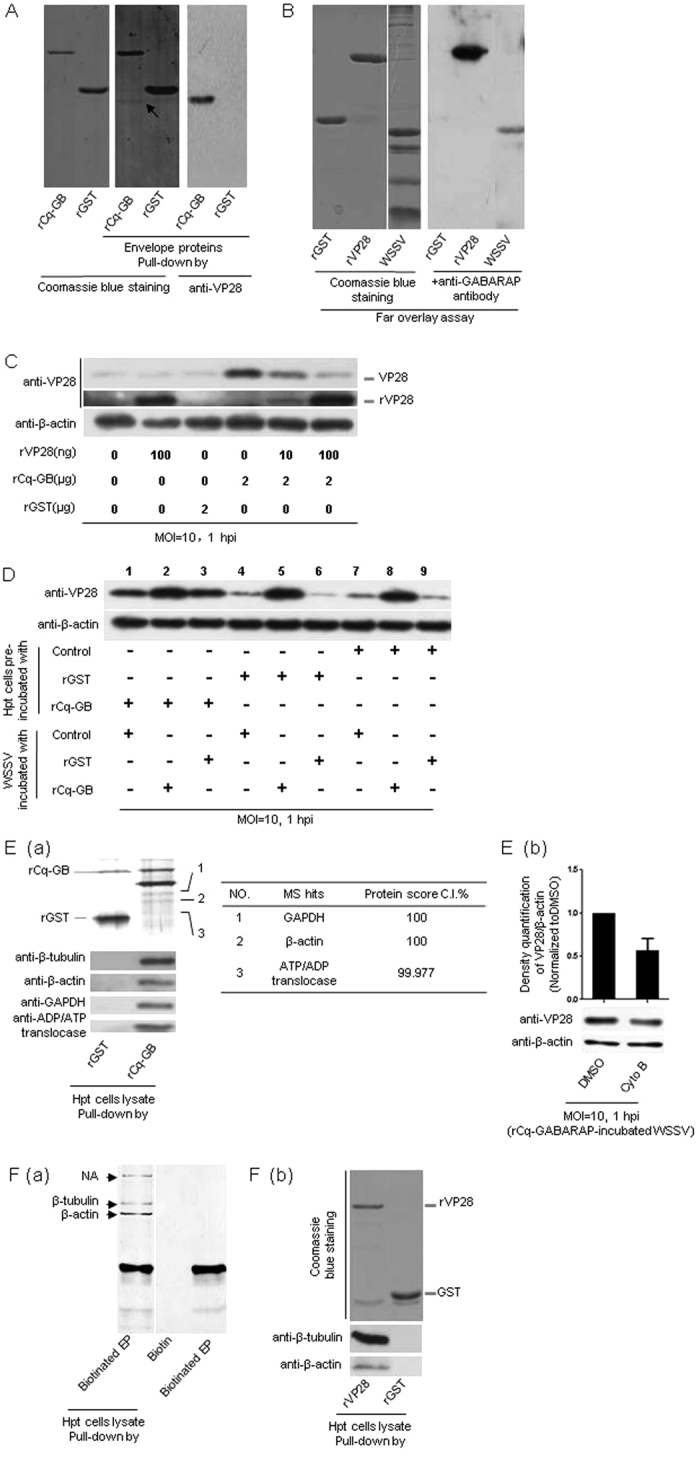
Protein interactions between rCq-GABARAP, WSSV and Hpt cell led to viral entry promotion. (**A**) rCq-GABARAP bound to the viral envelope protein VP28 in pull-down assays. The pull-down samples were resolved by SDS-PAGE followed by Coomassie blue staining (middle panel) and immunoblotting with an anti-VP28 monoclonal antibody (right panel). The arrow indicates the viral envelope protein VP28 which was further determined by MALDI TOF/TOF mass spectrometry. (**B**) rCq-GABARAP binding to the viral envelope protein VP28 confirmed by a far-western overlay blotting assay. The protein samples in the SDS-PAGE gel (left panel) were transferred to a PVDF membrane, which was then incubated with rCq-GABARAP and detected by immunoblotting with an anti-GABARAP antibody (right panel). (**C**) rCq-GABARAP-promoted WSSV entry attenuated by incubation with rVP28. (**D**) Synergistic effect on enhanced WSSV entry by rCq-GABARAP via targeting both WSSV and Hpt cell. (**E**) Interactions of rCq-GABARAP with the cytoskeleton components of Hpt cell. (a) The proteins pulled down from the Hpt cell lysate by rCq-GABARAP as detected by silver staining or Western blotting. (b) Suppression of rCq-GABARAP-mediated WSSV entry via pre-treatment of the cells with cytochalasin B as determined by immunoblotting to quantify the viral envelope protein VP28 (low panel). The band intensities of VP28 and β-actin from three independent experiments were analyzed using the Quantity One program (upper panel). (**F**) Interactions of WSSV with Hpt cell. (a) Binding of Hpt cell proteins to biotinylated WSSV envelope proteins (EP) was examined by pull down assay. The specific protein bands that only bound to the biotinylated envelope proteins indicated with arrows were selected for mass spectroscopy determination. NA, data not available. (b) Binding of β-tubulin or β-actin with rVP28. Hpt cell lysate was prepared and used for pull-down assays with rVP28 or GST as a control. The sample was analyzed using an anti-β-tubulin or anti-β-actin antibody. Note: The results in B and F(a) were assembled by rearranging and joining images of different lanes that originated from the same film. All results were observed in at least three independent experiments, and one set of representative results is shown.

In addition to its key role in autophagy, GABARAP is well known as a traffic protein by binding to membrane-associated proteins^22^,^35^,^36^, or as a scaffold protein in membrane targeting of the ubiquitin ligase which is critical for the regulation of Rac1 signaling^24^. Because rCq-GABARAP was able to promote WSSV entry into Hpt cell after its incubation with the virions, and Cq-GABARAP was co-localized with VP28 on the cell membrane of Hpt cell, we then asked whether rCq-GABARAP was capable of binding to any viral envelope protein, which might contribute to the observed enhancement of viral entry. As examined using a protein pull-down assay, rCq-GABARAP bound to the viral envelope protein VP28, as determined by an immunoblotting assay with an anti-VP28 antibody (Fig. 6A). This was further confirmed by the binding of rCq-GABARAP to rVP28 or VP28 from the mixture of the isolated viral envelope proteins in a far-western overlay blotting assay with an anti-GABARAP antibody (Fig. 6B), which clearly showed specific binding between Cq-GABARAP and VP28. Furthermore, the introduction of additional rVP28 into the pre-incubation of WSSV and rCq-GABARAP obviously reduced viral entry accompanied by increased cellular uptake of rVP28 (Fig. 6C). This finding suggested that the additional rVP28 could suppress the rCq-GABARAP-mediated enhancement of WSSV entry, possibly by competing for the binding of rCq-GABARAP with the VP28 on the virion envelope. To further elucidate whether the enhanced viral entry could also be achieved by introduction of extra-Cq-GABARAP into Hpt cell, the cells were pre-incubated with rCq-GABARAP followed by viral infection and then the assessment of viral entry. The data revealed a clear increase of WSSV entry into Hpt cell after pre-incubation with rCq-GABARAP compared with the rGST- or no-protein-incubated control cells (lane 1, 4 and 7 in Fig. 6D), suggesting that rCq-GABARAP probably promoted WSSV entry not only through direct binding to the virion envelope components, such as VP28 (Fig. 6A), but also by directly affecting the Hpt cell. Moreover, because rCq-GABARAP could significantly promote viral entry after binding to the virions (Fig. 4C), we then determined the effect of rCq-GABARAP on the rCq-GABARAP-mediated enhancement of viral entry by comparing its binding to WSSV and its impact on Hpt cell through a “double-incubation” assay, in which the WSSV and Hpt cell were both incubated with rCq-GABARAP before the assessment of viral entry. The results showed that the pre-incubation of WSSV with rCq-GABARAP (lane 2, 5 and 8 in Fig. 6D) significantly enhanced viral entry compared with the internal controls (Fig. 6D). Together, these findings demonstrated that rCq-GABARAP could promote WSSV entry by both binding to WSSV and affecting Hpt cell but achieved stronger viral entry promotion through its direct incubation with WSSV virions, probably by binding to VP28 as indicated above (Fig. 6C). Because WSSV entry was also promoted by pre-incubation of the Hpt cell with rCq-GABARAP (Fig. 6D), we next investigated the interaction of rCq-GABARAP with the Hpt cell by performing a protein pull-down assay to isolate the putative proteins that might bind to rCq-GABARAP with the aim of obtaining molecular insights into how rCq-GABARAP affected Hpt cell to promote the viral entry, for example whether rCq-GABARAP could interact with cytoskeleton in consideration to that the transport of GABA_A_ receptor and κ opioid receptor mediated by rCq-GABARAP is achieved by its interaction with cytoskeleton^35^,^36^. The results showed that rCq-GABARAP could bind to Cq-β-tubulin and Cq-β-actin (Fig. 6E(a)), which suggested that rCq-GABARAP probably promoted WSSV entry via interaction with the cytoskeleton of Hpt cell. Indeed, cytochalasin B, a microfilament inhibitor, suppressed the rCq-GABARAP-mediated enhancement of WSSV entry (Fig. 6E(b)), supporting the hypothesis that rCq-GABARAP promotes WSSV entry by cooperation with microfilaments. In contrast, the Hpt cell protein pull-down assay using biotinylated WSSV envelope proteins revealed that both Cq-β-tubulin and Cq-β-actin bound to the mixture of viral envelope proteins (Fig. 6F(a)). These binding events of Cq-β-actin and Cq-β-tubulin to VP28 were further confirmed by their binding to rVP28 with a single protein pull-down assay, respectively (Fig. 6F(b)). Taken together, these data implied that Cq-GABARAP may act as a traffic protein during viral entry and participate in intracellular viral transport by interacting with cytoskeleton.

### rCq-GABARAP suppressed WSSV replication

**Figure 7:**
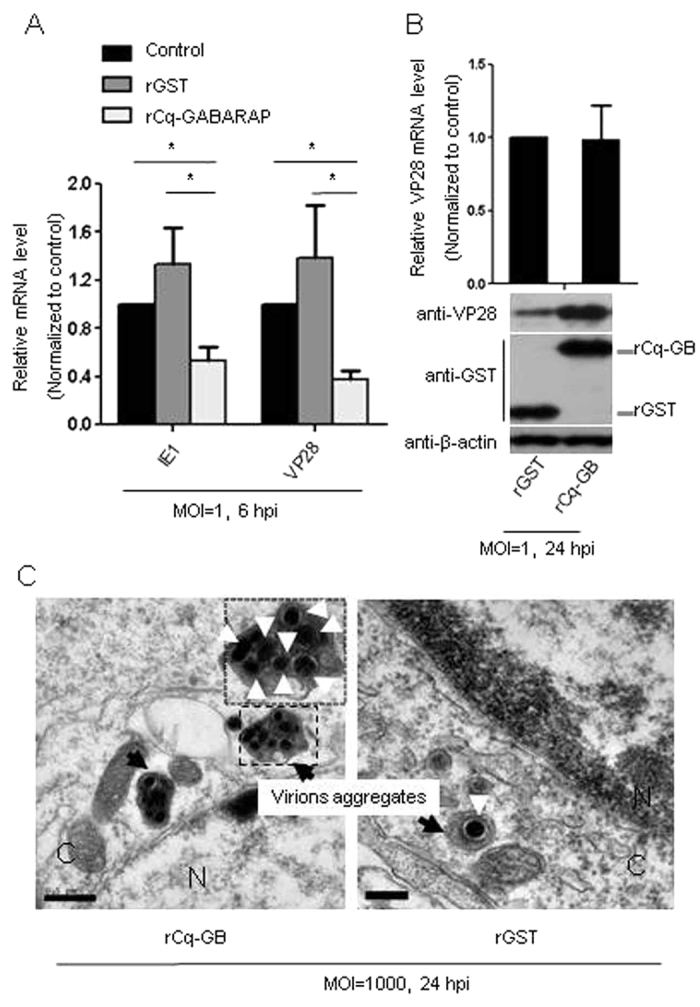
Suppression of WSSV replication by rCq-GABARAP. WSSV at different MOIs was incubated with rCq-GABARAP as indicated and then inoculated into Hpt cell cultures. (**A**) Quantification of WSSV transcripts (IE1 and VP28) by qRT-PCR. The Hpt cells were infected with virus pre-incubated with rCq-GABARAP or rGST, and the relative gene transcript expression of IE1 and VP28 was compared to that obtained with virus without incubation with the recombinant protein. (**B**) WSSV entry but not viral replication (upper panel) enhanced by rCq-GABARAP at a late stage of viral infection (24 h) (lower panel). Increased VP28 protein levels were detected in Hpt cell infected with WSSV pre-incubated with rCq-GABARAP as determined by immunoblotting against the viral envelope protein VP28 at 24 hpi. (**C**) An increased number of WSSV virion aggregates was found in the Hpt cell infected with WSSV that had been pre-incubated with rCq-GABARAP compared to those infected with the rGST pre-incubated virus as visualized by TEM. Bars: 0.5 μm in the left image, 0.2 μm in the right image; C, cytoplasm; N, nucleus. WSSV virions are indicated with white arrowheads. WSSV virion aggregates are indicated with black arrows. All of the results were observed in at least three independent experiments, and one set of representative results is shown. The data are presented as the mean ± SEM from at least three independent experiments and were analyzed by Student’s *t* test (*P < 0.05).

Because Cq-GABARAP was found to play an important role in promoting WSSV entry via both the CME pathway and autophagy pathway as described above, we then addressed whether it either promoted or suppressed viral replication after the enhanced viral entry. Using qRT-PCR, the transcription of the viral genes was significantly suppressed at 6 hpi after infection with the WSSV pre-incubated with rCq-GABARAP for 0.5 h (Fig. 7A). In addition, the transcript levels of VP28 were similar between the Hpt cell infected with rCq-GABARAP-incubated virus and the control virus at 24 hpi (upper panel in Fig. 7B), indicating no enhancement of viral replication by rCq-GABARAP treatment at this infection stage. However, the immunoblotting assay showed that increased VP28 protein was detected in Hpt cell infected with rCq-GABARAP-incubated virus at 24 hpi compared to the control treatment (lower panel in Fig. 7B), suggesting the increased internalization of WSSV but lower synthesis of new viral proteins, i.e., suppression of viral replication when pre-incubated with rCq-GABARAP. Furthermore, TEM analysis showed that larger WSSV aggregates were present at 24 hpi in Hpt cell infected with the virus pre-incubated with rCq-GABARAP compared with the cells infected with the virions pre-incubated with rGST (Fig. 7C). The number of WSSV virions in the aggregates was probably increased during the WSSV infection process as shown by TEM, implying that Cq-GABARAP might gradually “gather” individual virions or small viral inclusions to form larger viral aggregates intracellularly.

## Discussion

In the present study, we focused on the early viral entry events during WSSV infection in crayfish Hpt cell and found that multiple endocytic pathways, including CME, macropinocytosis and caveolae-mediated endocytosis, were all utilized for viral entry. CME is regarded as a vital endocytic route for the internalization of extracellular cargos that exists in most eukaryotic cells and is also widely recruited by many viruses for cellular entry^13^. The size of the clathrin-coated pits/vesicles (CCPs/CCVs) is typically approximately 60–200 nm, but this size can be increased as the result of recruitment by certain pathogens, such as vesicular stomatitis virus (VSV, 70 nm × 70 nm × 200 nm) and listeria (500 nm × 500 nm × 500–2000 nm), for internalization^14^. Here, we showed that the WSSV virions (70–167 nm × 210–380 nm) were clearly captured in invaginations, similar to VSV^26^, which enters the host cell via CME. Thus, this finding implied that WSSV entered the Hpt cell by forming larger CCPs, which is consistent with a recent study by Huang *et al*.^11^. During CCP formation, clathrin is usually recruited to coat and assemble on the cytosolic side of the cell membrane, which can be efficiently interrupted by CPZ treatment^27^. Although CPZ did not affect WSSV entry into shrimp hemocyte^7^ or crayfish granulocyte^6^, our present data and the recent study by Huang *et al*.^11^ together clearly showed that both WSSV entry and viral replication were markedly reduced by pretreating the cells with CPZ, suggesting that diverse routes were involved in WSSV entry into crayfish Hpt cell and hemocyte. Additionally, the inhibition of WSSV entry with dynasore both in our present study and Huang’s study^11^ together implied that WSSV entry was also dynamin-dependent. Furthermore, our study provided direct molecular evidence that the gene silencing of Cq-CLC, Cq-AP50 or Cq-dynamin, the key components of the CME machinery, reduced WSSV entry. Thus, these results provided a clear demonstration at the molecular level that CME is an important route for the entry of a crustacean virus WSSV into the host cells. Macropinocytosis, an endocytic process normally used for fluid internalization, has received increased attention due to its involvement in the entry of a growing number of viruses^30^. Krzyzaniak *et al*. found that the entry of respiratory syncytial virus (RSV) into HeLa cells via macropinocytosis was reduced by the inhibition of protein kinase C with rottlerin^37^. By targeting Na^+^/H^+^ exchangers, the EIPA inhibition assay is commonly used for defining macropinocytosis^30^. For example, the cellular entry of Ebola virus^38^ or foot-and-mouth disease virus^39^ into mammalian cells is sensitive to the presence of EIPA. Using TEM, we clearly showed that WSSV virions were captured by filopodia-like protrusions or macropinocytic cups, which are typical features of micropinocytosis for the first time^30^. In addition, WSSV entry was significantly suppressed by pharmacological inhibitors, such as rottlerin and EIPA, which are commonly used to block the action of macropinocytosis, but enhanced by the macropinocytosis inducer PMA. Thus, our findings suggested that WSSV entered Hpt cell via an alternative endocytic route by macropinocytosis. Furthermore, cholesterol functions as a modulator of membrane fluidity and a protein anchor (like caveolin) associated with caveolae-mediated endocytosis, and it is also can be required for clathrin-dependent endocytosis^40^ or macropinocytosis^41^. Caveolae-mediated endocytosis is characterized by the invagination of caveolae rich in cholesterol and this pathway is sensitive to MβCD, filipin or nystatin, which are widely used for depleting or binding to cholesterol and for disrupting caveolae function^42^. Two recent studies reported that MβCD treatment suppressed WSSV invasion in both shrimp^7^ and crayfish hemocyte^6^, which was in agreement with the observation on viral infection in crayfish Hpt cell both in our present study and that by Huang *et al*.^11^. In addition, Wang *et al*.^5^ claimed that caveolae-mediated endocytosis might be employed by WSSV to enter shrimp hemocyte via the depletion and replenishment of cholesterol on the cell membrane. As viral cholesterol is a crucial determinant for the infectivity of dengue virus^43^ and influenza virus^44^, any possible interference of viral envelope cholesterol should be excluded. In our present study, the medium was replaced with fresh medium after incubation with exogenous cholesterol, and WSSV entry was also clearly found to be restored by cholesterol replenishment. Thus, these results highlighted the essential role of cholesterol in the cell membrane for WSSV entry and suggested another alternative route for viral entry via caveolae-mediated endocytosis. This speculation was supported by the findings from Huang *et al*.^11^ showing that the tyrosine kinase inhibitor genistein specifically inhibited caveolae-mediated endocytosis and induced slight inhibition of WSSV internalization and infection.

Interestingly, our TEM analysis clearly showed the localization of WSSV virions within autophagosomes at 30 min (i.e., the viral entry stage of WSSV infection), which directly supported the hypothesis that autophagy was associated with WSSV entry into Hpt cell. This finding was similar to that obtained in a study of foot-and-mouth disease virus, which is able to induce the formation of autophagosomes and localizes to autophagosomes during its entry at the early stage of infection^45^. WSSV entry was increased by the induction of autophagy with rapamycin and decreased by the inhibition of autophagy with L-Asn, indicating that WSSV likely takes advantage of autophagy for its “entry” or early intracellular trafficking after across the Hpt cell membrane. Additionally, Su *et al*. recently found that WSSV infection could trigger the PI3K-Akt-mTOR pathway^46^, the well-known signaling pathway that controls the induction of autophagy^47^. Our present study further showed that the Cq-GABARAP was also positively associated with WSSV infection for its altered expression and distribution post viral challenge. GABARAP was originally found to bind to GABA_A_ receptors^48^ and to act in the trafficking of the GABA_A_ receptor to the cell membrane^49^. Multiple sequence alignments revealed that Cq-GABARAP shares 95% identity with human GABARAP (Supplementary Fig. 2) and belongs to the GABARAP subfamily of the Atg8 protein family. Importantly, the co-localization of Cq-GABARAP with VP28 on Hpt cell membranes strongly suggested that Cq-GABARAP played a vital role in viral entry. Furthermore, through inhibition of Cq-GABARAP and the addition of extra rCq-GABARAP, we demonstrated that Cq-GABARAP was necessary for promoting WSSV entry through direct binding with the viral envelope protein VP28, which could be reduced by the addition of rVP28 through protein competitive binding. Moreover, rCq-GABARAP was capable of binding to extra rVP28 and enhancing its cellular internalization, which in turn suppressed the rCq-GABARAP-mediated enhancement of viral entry in a dose-dependent manner. However, it was not clear how the binding of VP28 by rCq-GABARAP contributed to the “pumping” of WSSV virions from the extracellular space into the cytoplasm.

As an important biological process, CME needs to be precisely regulated by its cargo and PI(4,5)P2 on the cell membrane^50^. Because GABARAP can bind to CHC^23^, CLC (our unpublished data) or the typical cargo transferrin receptor, which uses CME for internalization^22^, we hypothesized that the Cq-GABARAP-related cellular events were strongly correlated with CME. Indeed, the promotion of WSSV entry by Cq-GABARAP was partially abolished by pretreatment with the CME inhibitor CPZ or gene silencing of Cq-AP50 or Cq-CLC, strongly supporting the hypothesis that the Cq-GABARAP-mediated enhancement of WSSV entry was dependent on CME. GABARAP is able to promote transferrin receptor internalization by repressing the Rac1 signaling pathway through targeting synaptojanin 2 to accelerate the circulation of PI(4,5)P2 and CCP formation in HeLa cells^20^. In our present study, the transfection of recombinant Cq-Rac1 protein, which may elevate Rac1 activity to suppress CME action, could inhibit WSSV entry (Supplementary Fig. 3), suggesting that the molecular regulation of CME by Rac1 is conserved from invertebrates to mammals. In addition, Cq-GABARAP-mediated WSSV entry promotion was enhanced by silencing of the Cq-Rac1 gene to abolish its proposed suppression of CME regulation^20^, implying that Cq-GABARAP might act as an upstream modulator by targeting the Rac1 signaling pathway to enhance CME-mediated uptake of WSSV.

GABARAP plays important roles in the intracellular transportation of proteins like GABA_A_ receptor^35^, κopioid receptor by interaction with cytoskeletal components, which was also observed in the crayfish Hpt cell because rCq-GABARAP bound to Cq-β-tubulin and Cq-β-actin. This interaction may contribute to its activity in the intracellular trafficking of WSSV in crayfish Hpt cell because the rCq-GABARAP-mediated enhancement of WSSV entry was repressed by the microfilament-disrupting agent cytochalasin B. Interestingly, in comparison to the effects on Hpt cell exerted by Cq-GABARAP via targeting Rac1 signaling and the cytoskeleton as mentioned above, the direct binding of rCq-GABARAP to virions was correlated with a much stronger promotion of viral entry. A possible explanation is that the virions might benefit from using the cytoskeleton for intracellular transportation due to the binding of rCq-GABARAP to both the virions and the cytoskeleton, whereas rCq-GABARAP may activate cellular signaling to promote viral entry by targeting Hpt cell, which is less efficient for promoting viral entry. In addition, the viral envelope protein VP28 was also found to bind Cq-β-tubulin and Cq-β-actin, highlighting the possibility that these cytoskeletal components were involved in the entry of WSSV. Interestingly, Cq-GABARAP also interacted with Cq-GAPDH and Cq-ATP/ADP translocase, both of which play vital roles in energy production to support the physical activity of the cells. Further investigations are required to determine whether the protein interactions between Cq-GABARAP and molecules related to energy maintenance in the Hpt cell also contribute to WSSV entry.

Surprisingly, the Cq-GABARAP-mediated enhancement of viral entry did not facilitate WSSV replication but significantly suppressed viral replication at 6 hpi, which is an early stage of infection. As the formation of neuraminidase-deficient influenza virus aggregates has been suggested to confine virion propagation and thus decrease virus infectivity^51^, the increased WSSV aggregation observed in the cytoplasm after rCq-GABARAP treatment might provide a possible explanation for the antiviral mechanism of Cq-GABARAP. However, WSSV replication was observed to be relatively equal between the rCq-GABARAP-incubated virus and the control treatment during the late stage at 24 hpi, suggesting that rCq-GABARAP might have a limited capacity to restrict viral replication in Hpt cell in contrast to its significant effect on the promotion of WSSV entry. Further studies examining the mechanism underlying WSSV aggregation via rCq-GABARAP, particularly the correlation with CME as well as the fate of the aggregated virions, are necessary to elucidate the intracellular antiviral role of Cq-GABARAP.

**Figure 8:**
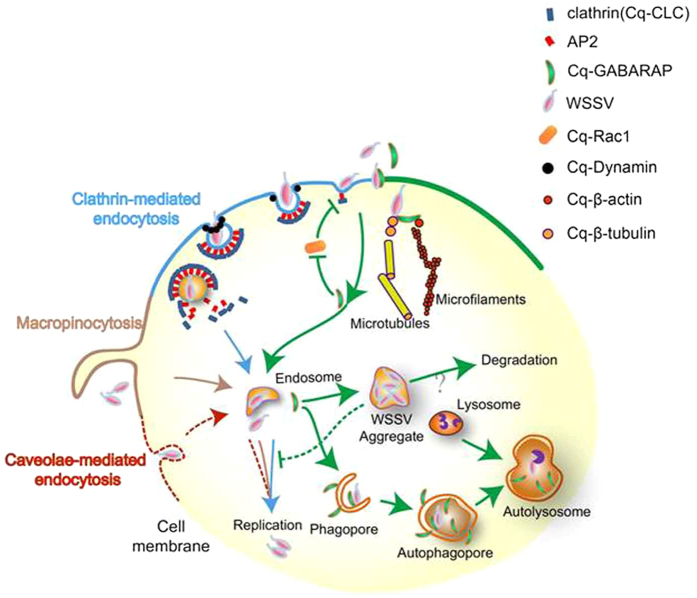
Proposed model for WSSV entry into crayfish Hpt cell. Based on the data from this study, CME, macropinocytosis and caveolae-mediated endocytosis are clearly employed for both WSSV entry and viral replication in Hpt cell. Cq-GABARAP, a key autophagy-related molecule that can bind to both WSSV and cytoskeleton components, is co-localized with the viral envelope protein VP28 on the cell membrane, leading to the promoted WSSV entry possibly by 1) binding to WSSV virions; 2) cooperating with cytoskeletal proteins such as β-actin and β-tubulin; 3) enhancing CME activity via suppression of the Rac1 signaling pathway; 4) increasing autophagic activity. However, WSSV replication is suppressed in the presence of rCq-GABARAP after its entry into Hpt cell, which may be attributed to the intracellular formation of WSSV aggregates that delay transport of virions from the cytoplasm into the nucleus for successful replication.

In summary, we propose a general hypothesis of WSSV entry into crayfish Hpt cell as illustrated in Fig. 8 based on our findings. WSSV takes advantage of multiple endocytic routes, such as CME, macropinocytosis and caveolae-mediated endocytosis. Interestingly, WSSV entry is also positively correlated with the versatile protein Cq-GABARAP, which targets a viral envelope protein to link the virions to cell membrane and further the cytoskeleton components in the Hpt cell, possibly by regulating endocytosis as well as autophagy. However, rather than promoting WSSV replication, Cq-GABARAP suppresses viral replication, which is probably associated with the formation of viral aggregates in the cytoplasm. Thus, further investigations of the key role of Cq-GABARAP in autophagy and in WSSV-host cell interactions will likely provide a better understanding of the novel events that occur during WSSV pathogenesis.

## Materials and Methods

### Animals

Freshwater red claw crayfish, *C. quadricarinatus*, were obtained from Xiamen Xinrongteng Aquaculture Technology Development Co., Ltd and Xianyou Tenglong Aquaculture Co. Ltd, China, and maintained in aerated tanks at room temperature. Only inter-molting healthy crayfish were used in this study.

### Hpt cell cultures and virus

Hpt cells were isolated from *C. quadricarinatus* and cultured as described by Söderhäll *et al*.^9^ (More detail can be found in supplementary information). WSSV was kindly provided by Prof. Xun Xu (Third Institute of Oceanography, SOA, Xiamen, Fujian, China). The virus was prepared as described by Xie *et al*. and quantified via absolute quantification by PCR^52^.

### Reagents

With the exception of nystatin and rottlerin, which were obtained from Inalco and Acros Organics, respectively, the chemicals were purchased from Sigma-Aldrich. The monoclonal antibody against VP28 was provided by Prof. Feng Yang (Third Institute of Oceanography, SOA, Xiamen, Fujian, China). The monoclonal antibodies against β-actin and GABARAP were purchased from ZSGBB (Zhongshan Golden Bridge Bio-technology, Beijing, China) and Eptomics, respectively. The monoclonal antibody against β-tubulin was obtained from MultiSciences (Lianke) Biotech Co., Ltd, China. HRP-conjugated secondary antibodies against mouse or rabbit IgG, Alexa Fluor 488-conjugated goat anti-rabbit IgG and Alexa Fluor 594-conjugated goat anti-mouse IgG were purchased from ZSGBB. The pGEX 4T-2/VP28 plasmid was a gift from Prof. Wen-lin Wu (Quanzhou Normal University, Quanzhou, Fujian, China).

### WSSV entry and replication assays

For WSSV replication determination, viral infection was performed by inoculating WSSV (MOI = 1, multiplicity of infection, i.e., the ratio of the virus to cell) into the Hpt cell cultures for 6 hpi before RNA isolation and qRT-PCR (See supplementary information). Similar infection assays were conducted in which the Hpt cells were infected with WSSV that had been pre-incubated with either 2 μg or the indicated amount of the recombinant proteins for 30 min. To determine WSSV entry, viral infection was performed as described above at an MOI of 10 for 1 h (or the indicated time intervals) before cell lysis in 10 μl of Laemmli sample buffer and immunoblotting analysis (See supplementary information).

### Hpt cell cultures treated with pharmacologic inhibitors

The Hpt cell cultures were incubated accordingly with modified L-15 medium containing 2.5 μM CPZ, 50 μM dynasore, 10 μM EIPA, 2 μg/ml filipin, 500 μM MβCD, 2 μg/ml nystatin, 1 μM rottlerin, 50 nM (or the indicated concentrations) rapamycin, 30 mM L-Asn, 5 μg/ml cytochalasin B or 2 μM PMA for 30 min before WSSV infection as described above. In the cholesterol replenishment experiments, the Hpt cell cultures were first treated with MβCD (500 μM) for 30 min. Then the medium containing MβCD was replaced with fresh L-15 medium containing 50 μM of water-soluble cholesterol and incubated for another 30 min. Afterwards, the Hpt cells were then incubated in fresh medium containing WSSV (MOI of 10) for 1 h before harvesting for immunoblotting as described in Supplementary information. Sterile water was used as a solvent control.

### RNAi assay

The RNAi assay was conducted using dsRNA. The dsRNA was synthesized using the MegaScript kit (Ambion, Austin, TX, USA) according to the manufacturer’s instructions and purified with the TriPure Isolation Reagent (Roche, USA). For dsRNA transfection, 250 ng of dsRNA/well (96-well plates) in 16 μl of RNase-free water was mixed with 0.75 μl of Cellfectin^®^ II Reagent (Life Technologies), maintained for 10 min at room temperature, appended with medium up to 100 μl and added into the cell wells containing 50 μl of medium. Half of the medium was replaced with fresh medium to reduce the toxic effects of Cellfectin after 3 h. The dsRNA transfection was repeated once one day after the first transfection as described above to improve RNAi efficiency. Viral infection was performed 24 h after the second dsRNA transfection as mentioned above. DsRNA generated from a GFP gene was also synthesized and used as a control treatment.

### Antibody transfection

The Hpt cell cultures were treated with an anti-GABARAP antibody delivered by PULSin (Polyplus, France), according to the manufacturer’s instructions. Briefly, the antibody was diluted in 20 mM HEPES and vortexed gently followed by the addition of PULSin and incubation at room temperature for 15 min, and then the mixture was inoculated into the cell cultures and incubated for 4 h. The WSSV was then added to the cell cultures and incubated for 1 h followed by wash once with the cell culture medium and cells collection for the determination of VP28 with immunobloting. Cells inoculated with BSA were used as the control treatment.

### TEM analysis

The Hpt cells were incubated with WSSV at an MOI of 1000, which is the typical MOI used for viral observation by TEM^53^, for 30 min or the indicated time interval. The cells were then washed with CPBS, harvested using a scraper, and centrifuged at 8,000 × *g* for 10 min. The cells were then fixed in 2.5% glutaraldehyde in CPBS at 4 °C for 2–4 h. The pellets were fixed in 1% osmium tetroxide for 2–3 h and washed again with CPBS. The fixed cells were dehydrated through an ethanol series followed by acetone and then embedded in Epon resin. The samples were sectioned with a microtome (Leica), and the sections were double-stained with uranyl acetate and lead citrate before their examination under a TEM (JEM2100HC).

### Labeling of WSSV with DiD

WSSV virions were freshly labeled with the lipophilic fluorescent dye DiD (4-chlorobenzenesulfonate salt; Life Technologies) as previously described^54^ with slight modifications. Briefly, the WSSV stock solution was mixed with DiD for 10 min and the free dye was removed using gel filtration columns (GE Healthcare) in HS buffer (2.5 mM HEPES, 145 mM NaCl). The Hpt cells were infected with DiD-WSSV (MOI of 20), and images were obtained and analyzed using an iCys laser-scanning cytometer (Beckman), which could automatically identify the target fluorescent signal events through proper contouring as described in the references^55^,^56^.

### Localization of Cq-GABARAP protein

To examine the intracellular distribution of Cq-GABARAP, 2.5 × 10^6^ Hpt cells were taken for cellular compartment isolation using the Proteoextract^®^ Subcellular Proteome Extraction kit (Millipore) according to the manufacturer’s instructions. The obtained proteins from different compartments, including cytoplasm, cell membrane and nucleus, were subjected to protein concentration determination followed by immunoblotting against Cq-GABARAP (see result in the Supplementary Fig. 2C). For the co-localization analysis of Cq-GABARAP with WSSV virions, an immunofluorescence assay was carried out. Briefly, Hpt cells were cultured on coverslips in a 24-well plate and fixed with 4% paraformaldehyde for 30 min followed by permeabilization with 0.1% Triton X-100 for 30 min. After blocking with 5% goat serum for 1 h, the Hpt cells were then washed three times with PBS and incubated with primary antibody against VP28 or GABARAP diluted in 0.1% goat serum (1:100) overnight at 4 °C. After washing three times with PBS, Alexa Fluor 488-conjugated goat anti-rabbit IgG or Alexa Fluor 594-conjugated goat anti-mouse IgG (1:200) was added and incubated for 2 h at room temperature. The nucleus was stained with DAPI, and cell imaging was performed with an LSM 780 confocal fluorescence microscope (Zeiss).

### Protein pull-down assays

To obtain the viral envelope proteins, 100 μl of 10^9^ copies/μl WSSV was solubilized by incubation with 0.5% Triton X-100 for 30 min at room temperature with gentle shaking as described by Xie *et al*.^52^. Briefly, after centrifugation at 20,000 × *g* and 4 °C for 30 min to pellet the viral nucleocapsid, the supernatant was transferred to a new tube as the viral envelope protein fraction. The envelope proteins were divided into two aliquots. One of the fractions was incubated with 2 μg of recombinant Cq-GABARAP protein (rCq-GABARAP) and 30 μl of glutathione Sepharose 4B resin with end-over-end mixing at 4 °C overnight. As a control, rGST was used in place of rCq-GABARAP. The samples were washed with PBS for at least seven times. The binding proteins were then resolved by 13.5% SDS-PAGE and subjected to Coomassie blue staining, silver staining, or immunoblotting to detect the WSSV envelope protein VP28. The specific protein band(s) that bound to rCq-GABARAP were excised for MALDI TOF/TOF mass spectrometric analysis. For the protein pull-down assays using Hpt cells, 100 mg of the freshly prepared crayfish hematopoietic tissue was ground in liquid nitrogen and then lysed in NP-40 containing a protease inhibitor cocktail (Roche). After incubation on ice for 40 min, the unresolved aggregates were removed by centrifugation at 15,000 × *g* for 20 min to generate a clear Hpt cell lysate. The Hpt cell lysate was subjected to a protein pull-down assay using the indicated bait proteins.

### Far-western overlay blotting assay

The protein samples were resolved by 13.5% SDS-PAGE and then transferred to a PVDF membrane. The membrane was then blocked with 5% skim milk in TBST for 2 h, briefly washed with TBST, and incubated overnight at 4 °C with the indicated recombinant proteins at a concentration of 25 nM. The membrane was then washed three times with TBST before immunoblotting detection with an anti-GABARAP antibody.

## Additional Information

**How to cite this article**: Chen, R.-y. *et al*. White spot syndrome virus entry is dependent on multiple endocytic routes and strongly facilitated by Cq-GABARAP in a CME-dependent manner. *Sci. Rep.* **6**, 28694; doi: 10.1038/srep28694 (2016).

## Supplementary Material

Supplementary Information
