# Supplementary material for: White spot syndrome virus entry is dependent on multiple endocytic routes and strongly facilitated by Cq-GABARAP in a CME-dependent manner
Source: Sci Rep. 2016 Jul 7;6:28694. doi: 10.1038/srep28694 (PMC4935888; doi:10.1038/srep28694)
Supplement: Supplementary Information [file srep28694-s1.pdf]

White spot syndrome virus entry is dependent on multiple endocytic routes and strongly facilitated by Cq-GABARAP in a CME-dependent manner

Rong-yuan Chen[1], Kai-li Shen[1], Zhen Chen[1], Wei-wei Fan[1, 3], Xiao-lu Xie[1], Chuang Meng[1], Xue-jiao Chang[1], Li-bing Zheng[1], Joseph Jeswin [1], Cheng-hua Li[3], Ke-jian Wang[1, 2] and Hai-peng Liu[1, 2*]

[1]State Key Laboratory of Marine Environmental Science, Xiamen University, Xiamen 361102, Fujian, PR China; [2]Fujian Collaborative Innovation Center for Exploitation and Utilization of Marine Biological Resources, State-Province Joint Engineering Laboratory of Marine Bioproducts and Technology , Xiamen 361102, Fujian, PR China; [3]Faculty of Life Science and Biotechnology, Ningbo University, 818 Fenghua Road, Ningbo 315211, Zhejiang, PR China[c].

[*]Address correspondence to Hai-peng Liu, Haipengliu@xmu.edu.cn. Tel.: +86 592 2183203; Fax: +86 592 2183203.

Running title: WSSV entry mechanism

Supplementary Figures 1 to 3, Table 1, and Methods

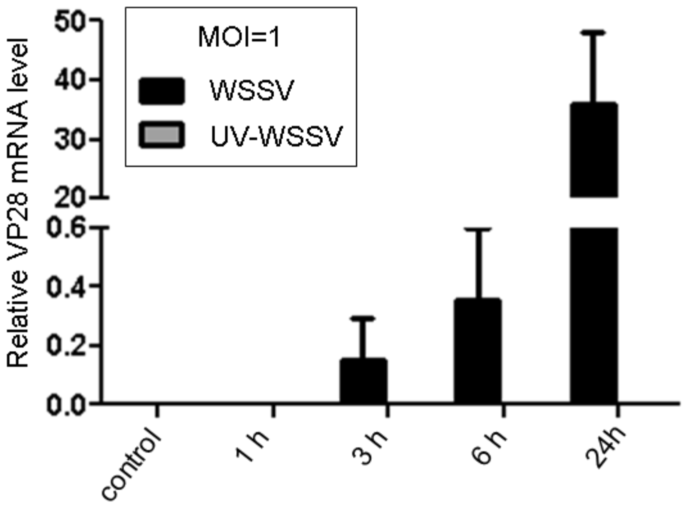

Supplementary Figure 1. Examination of the expression of the WSSV envelope protein VP28 during viral infection in Hpt cells by qRT-PCR. The Hpt cells were incubated with indicated virus inoculation, namely WSSV or UV-inactivated WSSV (UV-WSSV), or uninfected as control. After indicated time points (1, 3, 6 and 24 hpi), the cells were harvested for qRT-PCR, showing that no VP28 transcription was detected in Hpt cells uninfected or infected with UV-WSSV, while increased amount of VP28 translation since 3 hpi in the cells infected with WSSV.

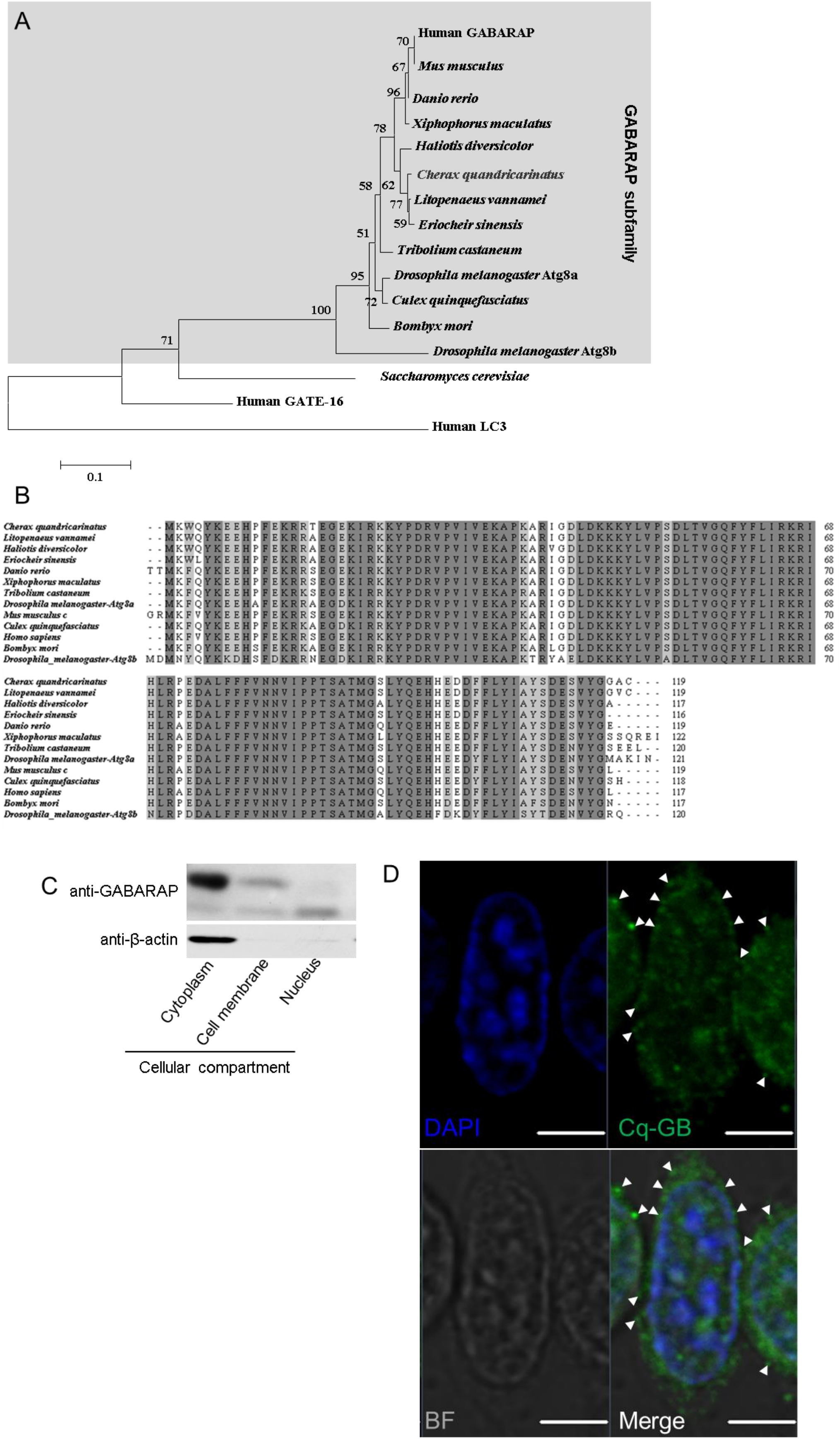

Supplementary Figure 2. Characterization of Cq-GABARAP. A: Phylogenetic

tree of Atg8 family proteins. B: Multiple sequence alignment of GABARAP from different species. The identical amino acid residues are dark gray, and similar amino acids are shown with a light gray background (*Litopenaeus vannamei*, AFV99179.1; *Haliotis diversicolor*, ADI56518.1; *Eriocheir sinensis*, AEE60804.1; *Danio rerio*, XP_003199067.2; *Xiphophorus maculatus*, XP_005804839.1; *Tribolium castaneum*, XP_973073.1; *Culex quinquefasciatus*, XP_001844428.1; *Drosophila melanogaster*-Atg8a, NP_727447.1; *D. melanogaster*-Atg8b, AAF55459.1; *Bombyx mori*, NP_001040244.1; *Mus musculus*, EDL12513.1; *Homo sapiens*, NP_009209.1; Human GATE-16, P60520; Human LC3, Q9H492; Human GABARAP, NP_009209.1). C: The distribution of Cq-GABARAP protein in different cellular compartments examined by immunoblotting. β-actin was used as a marker protein for the cytoplasmic compartment. D: Distribution of Cq-GABARAP in Hpt cells determined by IFA. Hpt cells were harvested for IFA analysis with anti-GABARAP antibody. The nucleus was stained with DAPI and cell images were taken under confocal microscopy. White arrowheads indicate some of the events that Cq-GABARAP located on cell membranes. Bars indicate 10 μm.

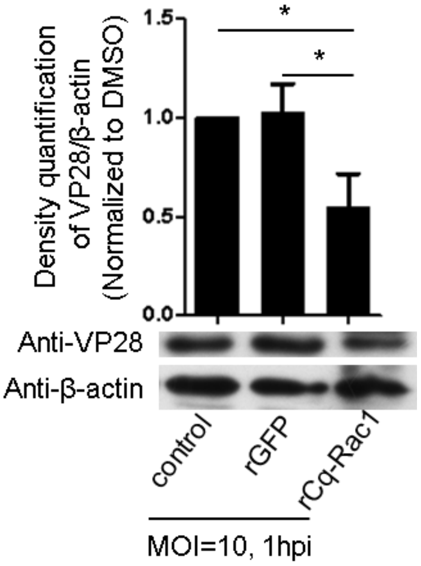

Supplementary Figure 3. Suppression of WSSV entry by recombinant Cq-Rac1 (rCq-Rac1). Hpt cell cultures were transfected with rCq-Rac1 (1 μg), rGFP or no protein as a control for 40 min using PULSin and then subjected to viral infection before viral entry examination by immunoblotting (lower panel). The band intensities of three independent experiments were analyzed using the QuantityOne program (upper panel). The data are presented as the mean ± SEM from at least three independent experiments and were analyzed by Student's *t* test (*$P<0.05$).

Supplementary Table 1. Primers used in the present study

Primers	Sequence information (5'–3')
**Gene clone**	
5’CLC 612-	CTTGATGGGAGGATTCTGCTTCA
3’CLC 210+	TTGAGATGCTGGGTGGAGATGA
Degenerate AP50+	TAYAAYCAYAARGGIGARGT
Degenerate AP50-	CCRAAYTTRCAYTCIGGCAT
5 AP50 357-	TGTGTCTGTGTTCTGAGGATACCCA
3 AP50 332+	TTGGGTATCCTCAGAACACAGACAC
5’GB 199-	ATGGTAGCGGAGGTTGGAGGAATGA
3’GB +	GTAGCGGAGGTTGGAGGGATGAC
Rac1+	CGAGGATGGAGGATTGTTGAG
Rac1-	TCGCTTGACTGGGAATCTGAG
5DYN 338-	TTCTGACCCGTAATGCGATCTGTG
3DYN 2085+	GCTCCTTGCCCACCTGTATGCC
**qRT-PCR**	
IE1-F	CTGGCACAACAACAGACCCTACC
IE1-R	GGCTAGCGAAGTAAAATATCCCCC
VP28-F	AAACCTCCGCATTCCTGT
VP28-R	GTGCCAACTTCATCCTCATC
16s-F	AATGCTTCTTGCACCACCAAC
16s-R	AGGTCTTGCTCAGCTGGATACC
Dete-CLC+	TGCTGAGGAGGTAAGCAAGATAG
Dete-CLC-	GCATTTAAGCCTTGATGGGAG
Dete-AP50 499+	AAGACAGCAAGCAAGGAGGAG
Dete-AP50 889-	CTGGAGGAACAAATGAAATAGAGTG
Dete-GB155+	CGTGCCGGTAATTGTAGAGAAGG
Dete-GB594-	AAACTGCACCAAGACACCCTG
dete Dyn 1853+	CATTCCTACGTGCTGGTGTTT
dete Dyn 2160-	TCGTTTACGTGCCTCCTCTGG
**RNAi**	
Ri-CLC+	TAATACGACTCACTATAGGGCGACCATCACACACCATCTACAC
Ri-CLC-	TAATACGACTCACTATAGGGTCTGTGCCTGGTTCCATCTTC
RiAP50 226+	TAATACGACTCACTATAGGGACGAACATTGCACGCACCA
RiAP50 669-	TAATACGACTCACTATAGGGCGCCACATGGGCAGACAA
Ri-GB 188+	TAATACGACTCACTATAGGGTCGCATTGGTGACTTGGATAAG
Ri-GB 639-	TAATACGACTCACTATAGGGGCAATTCTTTAGCACCTCAGGG
Ri Dyn 2055+	TAATACGACTCACTATAGGGCACGTCTATGGACCCTCAACT
Ri Dyn 2592-	TAATACGACTCACTATAGGGAAGAATCCCTGAATTACCCTGT
**Recombinant expression**	
eGB 1+	CGGGATCCATGAAGTGGCAGTACAAGG
eGB 396-	AAGCGGCCGCTCAGCACGCCCCTCCATA
eRac1 1+	GGAATTCCATATGATGTCATCTGGCCGTCCCAT
eRac1 588	CCGGAATTCTTAGAGGACTGAGCACTTGCGC

Supplementary Methods

Hpt cell cultures. The hematopoietic tissue from the dorsal side of the crayfish stomach was dissected, briefly washed with CPBS (crayfish phosphate buffer saline: 10 mM Na_2HPO_4, 10 mM KH_2PO_4, 150 mM NaCl, 10 mM $CaCl_2$, 10 mM $MnCl_2$ and 27 mM KCl, pH 6.8) and incubated in 500 μl of collagenase (0.1% collagenase type I and IV in CPBS) for 45 min at room temperature to dissociate the Hpt cells. The separated cells were washed twice with CPBS and then centrifuged for 3 min at 1,500 *g* and room temperature. The cell pellet was resuspended in modified L15 medium (Sigma-Aldrich) containing 5 μM 2-mercaptoethanol (Sigma-Aldrich), 1 μM phenylthiourea (Sigma-Aldrich), 60 μg/ml penicillin (Gibco), 50 μg/ml streptomycin (Gibco), 50 μg/ml gentamicin (Gibco) and 2 mM L-glutamine (Thermo Fisher) and then subsequently seeded at a density of 2×10^5 cells per 0.5 ml in 24-well plates. The prepared Hpt cells were allowed to attach for 30 min at room temperature, and 2 μl of a plasma preparation containing crude astakine was then added to each well as described by Söderhäll et al.[1]. One-third of the medium was replaced with fresh medium every second day.

Total RNA isolation and qRT-PCR: The Hpt cells were harvested for total RNA isolation using the GenElute™ Mammalian Total RNA Miniprep Kit (Sigma) at 6 hpi. After DNase I (Ambion) digestion, 1 μg of the purified total RNA was used for cDNA synthesis with the Prime Script™ RT Reagent kit (TaKaRa). qRT-PCR was performed, and the transcripts for IE1 (GenBank accession no. NC_003225.2) and VP28

(GenBank accession no. AF227911.1) were employed to assess the replication of WSSV in Hpt cell cultures. The crayfish 16S ribosomal RNA gene (GenBank accession no. AF135975.1)[2], a mitochondrial ribosomal RNA gene[3,4], was utilized as an internal control.

Immunoblotting analysis: The protein samples were separated by 13.5% SDS-PAGE and wet-transferred to a PVDF membrane. The membrane was blocked in 5% non-fat milk in TBST for 2 h, briefly washed with TBST, incubated with a 1:3000 dilution of the anti-VP28 primary antibody, and washed three times with TBST for 8 min/wash. The membrane was then incubated with an HRP-conjugated goat-anti-mouse IgG antibody at a dilution of 1:10,000 in TBST for 1 h, washed five times with TBST (8 min/wash) and detected using the ECL reagent (Minipore). The immunoblotting signal of protein bands was quantified with QuantityOne software (Bio-Rad).

Gene cloning: Cq-GABARAP, Cq-CLC, Cq-AP50, Cq-dynamin and Cq-RAC1. We previously isolated partial sequences of the Cq-GABARAP and Cq-CLC genes, which are transcriptionally up-regulated in Hpt cells following WSSV challenge[5]. Full-length cDNAs encoding the open reading frames of these genes were obtained by RACE-PCR. To clone other evolutionally conserved molecules involved in the CME process, such as Cq-AP50, Cq-dynamin and Cq-Rac1, degenerate PCR followed by RACE-PCR was performed to obtain the full-length cDNAs for these genes. All of the primers used for PCR are shown in Supplementary Table 1. The

BLAST (http://blast.ncbi.nlm.nih.gov/Blast.cgi) program was used to analyze the sequences, and Bioedit 7.0.9.0 and Mega 5.0 were used to conduct the multiple sequence alignments and phylogenetic analysis, respectively.

Preparation of the recombinant proteins. The open reading frame of Cq-GABARAP or Cq-CLC was cloned into the pGEX-4T-2 bacterial expression vector (GE Healthcare). The open reading frame of Cq-Rac1 or GFP was cloned into the pET28a bacterial expression vector (GE Healthcare) using primers as listed in Supplementary Table 1. The correct orientation and sequence of the cloned genes were confirmed by sequencing. The resulting plasmids were then introduced into *E. coli* (BL21), and IPTG was used to induce the ectopic expression of the inserted genes. The recombinant GST-fusion proteins were purified using an ÄKTA system with a GSTrap FF column (GE Healthcare) followed by dialysis against phosphate buffered saline (PBS, pH 7.4) to remove the excess GSH. The concentration of the resulting proteins was then determined, and the proteins were mixed with an equal volume of glycerol and stored at -80 ℃ for later use.

Biotinylation of WSSV: To isolate the putative viral binding proteins from the Hpt cells, the WSSV envelope proteins were biotinylated by incubating the freshly prepared viral envelope proteins with NHS-D-biotin (Sigma) at room temperature for 4 h. Ammonium chloride (NH_4Cl) was then added to the reaction solution to stop the labeling reaction, and the mixture was then dialyzed three times with PBS buffer (12

h/time) at 4 °C to remove the excess biotin. The obtained biotinylated viral envelope proteins were used for the pull-down assays.

References

1 Söderhäll, I., Kim, Y. A., Jiravanichpaisal, P., Lee, S. Y. & Söderhäll, K. An ancient role for a prokineticin domain in invertebrate hematopoiesis. *J Immunol* **174**, 6153-6160, (2005).

2 Crandall, K. A., Harris, D. J. & Fetzner, J. W., Jr. The monophyletic origin of freshwater crayfish estimated from nuclear and mitochondrial DNA sequences. *Proc Biol Sci* **267**, 1679-1686, (2000).

3 Yang, L. *et al.* Species identification through mitochondrial rRNA genetic analysis. *Sci Rep* **4**, 4089, (2014).

4 Kearsey, S. E. & Craig, I. W. Altered ribosomal RNA genes in mitochondria from mammalian cells with chloramphenicol resistance. *Nature* **290**, 607-608, (1981).

5 Liu, H.-P., Chen, R.-Y., Zhang, Q.-X., Peng, H. & Wang, K.-J. Differential gene expression profile from haematopoietic tissue stem cells of red claw crayfish, *Cherax quadricarinatus*, in response to WSSV infection. *Dev Comp Immunol* **35**, 716-724, (2011).
